# Uncovering the genomic basis of symbiotic interactions and niche adaptations in freshwater picocyanobacteria

**DOI:** 10.1186/s40168-024-01867-0

**Published:** 2024-08-10

**Authors:** Hongjae Park, Paul‑Adrian Bulzu, Tanja Shabarova, Vinicius S. Kavagutti, Rohit Ghai, Vojtěch Kasalický, Jitka Jezberová

**Affiliations:** Institute of Hydrobiology, Biology Centre of the Czech Academy of Sciences, České Budějovice, Czech Republic

**Keywords:** Picocyanobacteria, Freshwater, Genome collection, Symbiotic interaction, CRISPR-Cas

## Abstract

**Background:**

Picocyanobacteria from the genera *Prochlorococcus*, *Synechococcus*, and *Cyanobium* are the most widespread photosynthetic organisms in aquatic ecosystems. However, their freshwater populations remain poorly explored, due to uneven and insufficient sampling across diverse inland waterbodies.

**Results:**

In this study, we present 170 high-quality genomes of freshwater picocyanobacteria from non-axenic cultures collected across Central Europe. In addition, we recovered 33 genomes of their potential symbiotic partners affiliated with four genera, *Pseudomonas*, *Mesorhizobium*, *Acidovorax*, and *Hydrogenophaga*. The genomic basis of symbiotic interactions involved heterotrophs benefiting from picocyanobacteria-derived nutrients while providing detoxification of ROS. The global abundance patterns of picocyanobacteria revealed ecologically significant ecotypes, associated with trophic status, temperature, and pH as key environmental factors. The adaptation of picocyanobacteria in (hyper-)eutrophic waterbodies could be attributed to their colonial lifestyles and CRISPR-Cas systems. The prevailing CRISPR-Cas subtypes in picocyanobacteria were I-G and I-E, which appear to have been acquired through horizontal gene transfer from other bacterial phyla.

**Conclusions:**

Our findings provide novel insights into the population diversity, ecology, and evolutionary strategies of the most widespread photoautotrophs within freshwater ecosystems.

Video Abstract

**Supplementary Information:**

The online version contains supplementary material available at 10.1186/s40168-024-01867-0.

## Background

Picocyanobacteria, with a cell size of less than 3 µm, represent the smallest and most widespread phytoplankton in marine and freshwater ecosystems [1, 2]. This group of bacteria is affiliated with three distinct genera: *Prochlorococcus*, *Synechococcus*, and *Cyanobium* [1]. Currently, *Synechococcus* and *Cyanobium* have been further subdivided into three distinct phylogenetic groups referred to as subclusters (SC) 5.1 to 5.3 [3]. SC5.1 shows a close phylogenetic relationship with *Prochlorococcus*, collectively forming a marine-specific cluster of picocyanobacteria [4]. In contrast, strains from SC5.2 and 5.3 can inhabit diverse salinity gradients, including marine, brackish, and freshwater ecosystems [5]. SC5.3 is predominantly found in temperate and oligotrophic waterbodies [4, 6], while SC5.2 demonstrates remarkable adaptability to various environmental conditions [5].

The evolution of light-harvesting complexes, known as phycobilisomes, plays a crucial role in developing diverse pigmentations in picocyanobacteria and their adaptation to different light regimes [7]. Several studies of marine *Prochlorococcus* have unveiled ecologically significant ecotypes associated with growth elements such as iron [8], nitrogen [9], and phosphorus [10]. The recent discovery of novel *Synechococcus* strains in the deep oxygen-depleted water layers of the Black Sea demonstrated their potential to survive even in extreme conditions [11]. However, the genetic foundation behind their global success largely remains unresolved. The investigation of freshwater picocyanobacteria has faced even greater challenges, primarily due to the significant undersampling of their populations [12]. The recent release of a large genome collection for freshwater picocyanobacteria, consisting of 58 isolates, marks a significant advancement in the field [5, 13, 14]. However, most of these isolates originated from oligo- and mesotrophic lakes, indicating that their populations in various inland waterbodies might still be overlooked.

In shallow, eutrophic waterbodies, bacteria often encounter elevated mortality pressure from grazers (e.g., heterotrophic nanoflagellates) and viral infections [15], compared to their counterparts in deep, oligotrophic lakes. As protistan grazing is primarily cell size-dependent, both picocyanobacteria and heterotrophic bacteria can develop grazing-resistant forms, such as microcolonies, large aggregates, and filaments [16, 17]. Additionally, as part of their antiviral defense mechanisms, CRISPR-Cas (clustered regularly interspaced short palindrome repeats-CRISPR associated proteins) provides sequence-specific adaptive immunity for a wide range of bacteria and archaea [18]. However, there has been a prevailing consensus that CRISPR-Cas systems are rare in picocyanobacteria [19]. Notable exceptions to this consensus include *S. lacustris* Tous, *Vulcanococcus limneticus*, and a few other freshwater isolates [5], as well as *Synechococcus* sp. WH 8016 from the marine environment [6]. The absence of CRISPR-Cas systems in picocyanobacteria could potentially be attributed to their small genome sizes and the likelihood of employing alternative defense mechanisms that impose a lesser genetic load [19].

In the cultures of cyanobacteria where no carbon source is present, heterotrophic bacteria are often co-isolated as their symbiotic partners [20]. The strong interdependencies between them make acquiring and maintaining axenic cultures of picocyanobacteria a challenging task [21, 22]. Conversely, these non-axenic cultures offer a valuable opportunity to explore the unique microbial communities in their environment, as well as the metabolic interactions between hetero- and photoautotrophs. The diversity profiles of microbial communities associated with *Synechococcus* are influenced by the extent of ecological interactions and species coexistence [23]. The “helper” heterotrophic bacteria can benefit from phytoplankton-derived organic compounds while supporting enhanced growth or prolonged survival in nutrient-depleted conditions [20, 24]. These symbiotic interactions may involve nutrient cycling [22], vitamin trafficking [25], and removing reactive oxygen species (ROS) [25]. However, the mechanisms behind the assembly of heterotrophs in freshwater picocyanobacterial cultures and their symbiotic roles have never been reported.

To fill these gaps in our knowledge, we obtained non-axenic picocyanobacterial cultures from diverse freshwater ecosystems. This allowed us to recover the largest genome collection of freshwater picocyanobacteria, along with their abundances and cellular phenotypes under different conditions. Furthermore, we acquired the genomes of co-occurring heterotrophic bacteria present in the cultures. Our investigation into the global phylogeography of freshwater picocyanobacteria revealed ecologically significant ecotypes, associated with diverse environmental settings. Moreover, our genomic data provided insights into the symbiotic interactions of picocyanobacteria with heterotrophic partners and their adaptation strategies across different ecological niches.

## Materials and methods

### Sample collection, isolation, and culture

Picocyanobacterial isolates were obtained from water samples collected in 2005, and from additional samplings conducted between 2018 and 2020. During the sampling campaign in 2005, the isolation process comprised the following steps. Initially, water samples underwent filtration through 5-µm polycarbonate membrane filters (Sterlitech, Kent, WA, USA) to remove bigger organisms. The filtered samples were then processed by passing through 0.22-μm polyethersulfone membrane filters (Millipore, Merck, Darmstadt, DE) to enrich the picocyanobacterial biomass. Subsequently, the collected biomass was plated on BG11 medium solidified with 1.5% agar. Finally, individual colonies were picked and transferred into liquid BG11 medium, resulting in a total of 47 cultures from 17 different localities.

During the years between 2018 and 2020, we employed the dilution-to-extinction cultivation method to obtain isolates. Environmental samples were initially filtered through 0.1- or 0.2-µm polycarbonate membrane filters (Sterlitech, Kent, WA, USA), and enumerated using epifluorescence microscopy (Zeiss Imager.Z2, Carl Zeiss, Oberkochen, DE). After a serial dilution, the water samples were inoculated into 96-well plates containing 1.5 mL of BG11 or WC medium at an estimated 0.5 cell well^−1^. Cultures were incubated at room temperature for 3 weeks and evaluated for growth using epifluorescence microscopy (Zeiss Imager.Z2, Carl Zeiss, Oberkochen, DE). A total of 107 non-axenic cultures from 31 localities were obtained. All isolates were continuously cultivated in liquid WC media, ensuring that the reinoculation timespan did not exceed 2 months. While the majority (92.4%) of our picocyanobacterial cultures were unialgal, they were not axenic. Two or more picocyanobacterial genomes were acquired from 13 cultures (7.6%).

### Probe design and catalyzed reporter deposition-fluorescence in situ hybridization (CARD-FISH) analysis

To design ecotype-specific probes, 16S rRNA gene sequences obtained in this study and collected from publicly available databases were aligned using the MAFFT algorithm [26] within the Geneious software (https://www.geneious.com). Selected regions (18–25 nucleotides) were examined for hairpins and self-dimerization and evaluated in silico by the TestProbe application (https://arb-silva.de/search/testprobe) for hits outside the target group. Competitor oligonucleotides were designed when necessary and tested similarly. The mathFISH software (https://mathfish.cee.wisc.edu) was employed to determine the theoretical best hybridization conditions, and the final formamide concentration was determined in the lab. The list of designed probes and hybridization conditions are presented in Table S9.

Environmental samples (2 to 20 mL) were fixed with a final concentration of 2% formaldehyde for at least 2 hours and filtered on 0.2-µm pore-size polycarbonate filters (Millipore, Merck, Darmstadt, DE). A CARD-FISH protocol [27], with some modifications [28], was used for the labeling of distinct picocyanobacterial groups. Fluorescein-labeled thyramid, whose emission spectrum does not overlap with picocyanobacterial autofluorescence, was used for the amplification step. Filters were counterstained with DAPI and analyzed using epifluorescence microscopy (Zeiss Imager.Z2, Carl Zeiss, Oberkochen, DE) equipped with a Colibri LED light system. The images were captured using an Axiocam 506 (Carl Zeiss, Oberkochen, DE) with the following filter sets: DAPI 49 (Excitation 365; Beamsplitter TFT 395; Emission BP 445/50), fluorescein 38 HE (Excitation BP 470/40; Beamsplitter TFT 495; Emission BP 525/50), and chlorophyll *a* 62 HE (Excitation BP 370/40, 474/28, 585/35; Beamsplitter TFT 395 + 495 + 610; Emission TBP 425 + 527 + LP615 HE).

### Genomic DNA extraction

For genome sequencing, picocyanobacterial cultures in the stationary phase were centrifuged at 4 °C, 8000 rcf for 30 min to collect cell pellets, which were subsequently stored at − 80 °C until further processing. DNA extraction was carried out using the Quick-DNA Microprep Kit (Zymo Research, Irvine, CA, USA), according to the manufacturer’s instructions.

### Sequencing, preprocessing, and assembly of the sequencing reads

Genomic DNA samples were sequenced under the Illumina Novaseq 6000 platform (Novogene, Hong Kong, China), targeting 1 Gbp per sample as output. Low-quality reads were trimmed using reformat.sh and bbduk.sh (Phred score = 18) of the bbmap package (https://sourceforge.net/projects/bbmap/). Any adapters or PhiX contamination were eliminated using the bbduk.sh script. The preprocessed reads were de novo assembled using MEGAHIT v1.1.4 [29], employing k-mer sizes ranging from 29 to 149 in increments of 10. Finally, all assemblies underwent length-filtering, retaining contigs with a minimum size of 3 kbp.

### Recovery of the genomes and functional annotations

Since all the cultures we obtained were not axenic, genome binning was done for each assembly using MaxBin v2.2.7 [30], Metabat1 v2.15 [31], and Metabat2 [32], with default settings. Mean base coverage for each contig was generated by mapping preprocessed reads to the length-filtered assemblies using bbwrap.sh (kfilter = 31, subfilter = 15, maxindel = 80). Contig abundance files were obtained using jgi_summarize_bam_contig_depths [32] for Metabat1 and 2, and CoverM using trimmed_mean mode (https://github.com/wwood/CoverM) for MaxBin. Bins produced by each method were combined, dereplicated, and refined using DASTool v1.1.2 [33]. A taxonomy-based decontamination step was performed in order to produce high-quality bins: protein-coding genes from each contig were predicted using Prodigal v2.6.3 [34] in the metagenomic mode, and the taxonomy of each gene was assigned using MMseqs2 [35] with the GTDB r95 database [36]. Contigs with > 30% of genes without hits or hits to eukaryotes or viruses, as well as contigs in which the taxonomy disagreed with the consensus class, were removed. The resulting bins were further evaluated with CheckM v0.8.1 [37], and only those with ≥ 80% completeness and ≤ 5% contamination were retained for further analysis. Bins were finally renamed according to the sample of origin, method of binning (mx = Maxbin, m1 = Metabat1, m2 = Metabat2) and bin number. The taxonomy of the individual bin was assigned using GTDB-Tk v1.4.1 [38] and the GTDB r95 database [36]. The assignment of KO (K number) was done using KEGG-Orthology-And-Links-Annotation (KOALA) algorithm against the nonredundant KEGG Genes database [39].

### Phylogenomic analysis

A maximum-likelihood phylogenomic tree was constructed for the recovered picocyanobacterial genomes, as well as the references. All the genomes were scanned with hmmsearch for 120 conserved protein HMM markers [40]. Protein sequences for each maker were aligned using MAFFT v7.453 [26] in L-INSI mode and trimmed by trimAl v1.4 [41] with the following parameters: -gt 0.5 -keepheader. Subsequently, the trimmed alignments were concatenated using the catfasta2phyml.pl (https://github.com/nylander/catfasta2phyml), and used as input for tree construction using IQ-TREE2 v2.2.0 with 1000 iterations of ultrafast bootstrapping [42] and SH testing [43]. The best-fitting evolutionary model (Q.pfam+I+G4) was selected based on the BIC score by ModelFinder [44]. The final tree was visualized in the interactive Tree Of Life (iTOL) v6 (https://itol.embl.de/).

### Metagenomic read recruitment

A total of 724 publicly available metagenomes were subsampled to 20 million reads and used for read recruitment [45]. Prior to recruitment, rRNA genes in the individual genomes were masked. MMseqs2 [35] was used with the following parameters to align the sequencing reads to individual genomes and to calculate base coverage per Gb: -minid 0.95 -mincov 0.9 -minlen 50. To examine the presence of heterotrophs in our non-axenic cultures, we conducted the read recruitment of the genomes against the sequencing reads of the cultures using the same parameters. If the genome coverage of the sequencing reads was above 95%, we considered the genome to be present within the culture. Heatmaps were generated using the R package pheatmap v1.0.12 (https://github.com/raivokolde/pheatmap). Heat trees were generated using the R package metacoder v0.3.6 [46].

### Scanning and subtyping of CRISPR-Cas systems

We analyzed CRISPR-Cas systems in freshwater picocyanobacterial genomes, along with marine picocyanobacterial genomes (*n* = 97) from the Cyanorak database [47]. Additionally, we examined a collection of freshwater prokaryotic genomes (*n* = 9374) constructed in a previous study [48]. The CRISPRCasTyper v1.6.4 (https://github.com/Russel88/CRISPRCasTyper) was used for the scanning and subtyping of CRISPR-Cas genes. Plots were generated using the R package ggplot2 v3.3.5 [49].

### Phylogenetic tree reconstruction for Cas1

For the phylogenetic reconstruction of Cas1, we used all putative Cas1 sequences from the Uniprot database (https://www.uniprot.org/) and Cas1 predicted from a freshwater genome collection [48]. We manually curated the collected dataset by conducting a scan with hmmsearch [40] to identify significant hits associated with the Cas1 PFAM domain (PF01867). Hits with *p*-values less than 0.01 and sequence lengths exceeding 80 amino acids were retained. To further narrow down the selection, we opted for the top 10 hits for each picocyanobacterial Cas1 using MMseqs2 [35] (--cov-mode 0). The remaining sequences were clustered using MMseqs2 (easy-cluster workflow) with a minimum sequence identity of 90%. The resulting 416 Cas1 sequences were aligned with ﻿MAFFT v7.453 [26]. A Maximum-likelihood tree was generated with IQ-TREE2 [43] with the following parameters: --perturb 0.2 --nstop 500 -B 1000 -m TEST --alrt 1000.

## Results

### Phylogenomic overview and delineating ecologically significant ecotypes

**Fig. 1:**
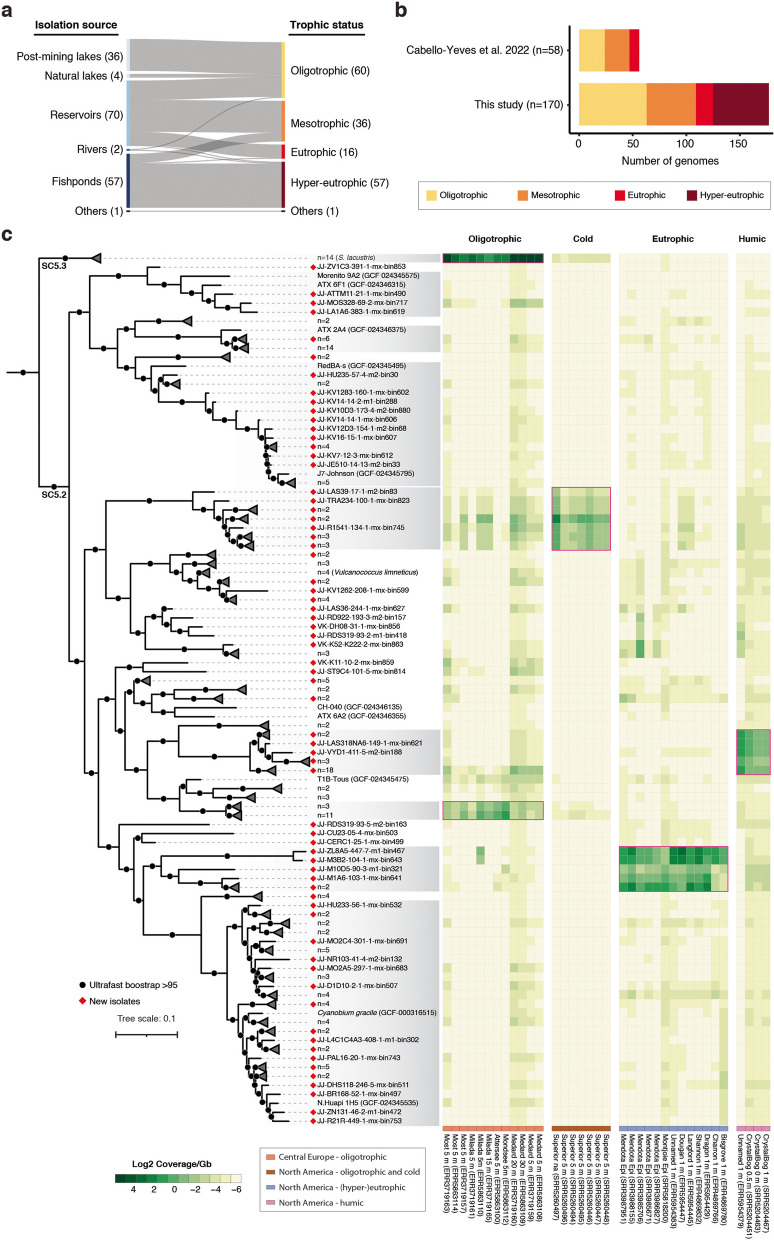
Isolation of picocyanobacterial genomes and delineating ecologically significant ecotypes.** a** Distribution of the picocyanobacterial genomes (*n* = 170) across different freshwater sources (lakes, reservoirs, rivers, and fishponds) and trophic status (oligotrophic to hyper-eutrophic). The number of the genomes is given in parentheses. **b** The number of newly obtained genomes compared to a prior study [13]. **c** Maximum likelihood phylogeny of freshwater picocyanobacterial genomes (*n* = 232) and their global distribution patterns (coverage per Gb). The genomes are collapsed to the species level (95% ANI cutoff), and the genomes within potential population boundaries (85% ANI cutoff) are marked by gray boxes. The individual or collapsed tree branches that are unique to the newly obtained isolates from this study are indicated with red diamonds. A complete tree including marine and brackish strains is available in Fig. S2. The complete dataset for the metagenomic read recruitment can be found in Table S3

We conducted a 3-year sampling campaign covering 44 different locations across Central Europe (Fig. S1 and Table S1). Our sampling approach was aimed to capture the diversity of picocyanobacterial population in freshwater ecosystems, including natural and post-mining lakes, reservoirs, rivers, and fishponds under different trophic statuses (Fig. 1a). This extensive sampling effort yielded 170 high-quality (≥ 80% completeness and ≤ 5% contamination) picocyanobacterial genomes from 156 non-axenic cultures (Table S2). Based on the trophic status of the isolation sources, these newly obtained genomes were initially classified into oligotrophic (*n* = 60), mesotrophic (*n* = 36), eutrophic (*n* = 16), and hyper-eutrophic (*n* = 57) groups (Fig. 1b). To provide a more comprehensive perspective for our study, we analyzed our new dataset alongside 79 previously published genomes of picocyanobacteria [6, 13, 50, 51]. A phylogenomic analysis affiliated the newly obtained genomes with either SC5.2 (92.4%; *n* = 157) or SC5.3 (7.6%; *n* = 13) (Fig. 1c and S2). The strains from SC5.3 showed the smallest average genome sizes (2.3 Mb) and lowest GC contents (51.8%), whereas those in SC5.2 exhibited a degree of flexibility, with genome sizes ranging from 1.9 to 4.2 Mb and GC contents from 55.5 to 72.5% (Fig. S3).

Potential population boundaries among these genomes were observed based on pairwise average nucleotide identity (ANI) at a cutoff above 85% (Fig. S4). Read recruitment in metagenomic samples (*n* = 724) further revealed the global distribution patterns of the isolated picocyanobacteria (Fig. 1c and Table S3). In line with previous studies [3], all the strains from SC5.3 were restricted to oligotrophic conditions. Among the members of SC5.2, we delineated ecologically significant ecotypes occupying four major freshwater regimes: oligotrophic, oligotrophic and cold (e.g., Lake Superior), eutrophic or hyper-eutrophic (e.g., Lake Mendota), and humic including environments displaying low pH (e.g., Lake Crystal Bog) (Fig. 1c). We designated these ecotypes as the low-nutrient (LN), low-nutrient and low-temperature (LNLT), high-nutrient (HN), and low-pH (LP) ecotypes, respectively, and employed these classifications for further analyses.

### Genome-resolved symbiotic interactions with co-occurring heterotrophs

**Fig. 2:**
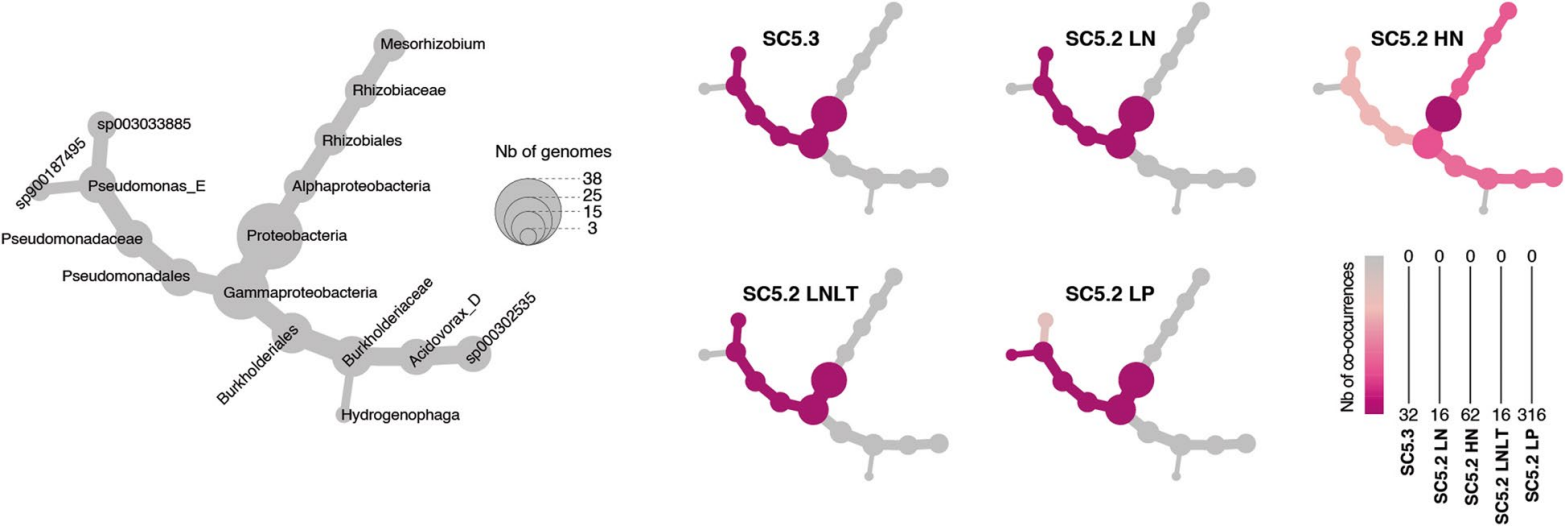
Heat trees for the co-occurrence pattern of the 38 heterotrophic bacterial genomes in picocyanobacterial cultures. The gray tree on the left functions as a key for the smaller unlabeled trees. Node sizes indicate the number of genomes for the respective bacterial taxa. Colors indicate the number of co-occurrences between heterotrophic bacteria and distinct picocyanobacterial ecotypes

Despite the absence of a major carbon source apart from vitamins and trace elements in the culture media, we additionally acquired 526 genomes of co-occurring heterotrophic bacteria, scoring ≥ 80% completeness and ≤ 5% contamination (Table S4). Among these, we revealed the non-random occurrences of 38 heterotrophs, repeatedly present in more than 5% of the cultures (Table S5). These potential symbionts belonged to taxonomically narrow lineages, affiliated with five different species within four genera, *Pseudomonas*, *Mesorhizobium*, *Acidovorax*, and *Hydrogenophaga*. The observed co-occurrences of picocyanobacteria and heterotrophs further provided insights into the selective association between them (Fig. 2). SC5.3, SC5.2 LN, and LNLT co-occurred with *Pseudomonas* (sp003033885) as their exclusive heterotrophic partner, while SC5.2 LP exhibited a strong preference for another *Pseudomonas* species (sp900187495). On the other hand, SC5.2 HN displayed a more complex association with heterotrophic partners, co-occurring primarily with *Mesorhizobium*, followed by *Acidovorax* and *Pseudomonas*.

**Fig. 3:**
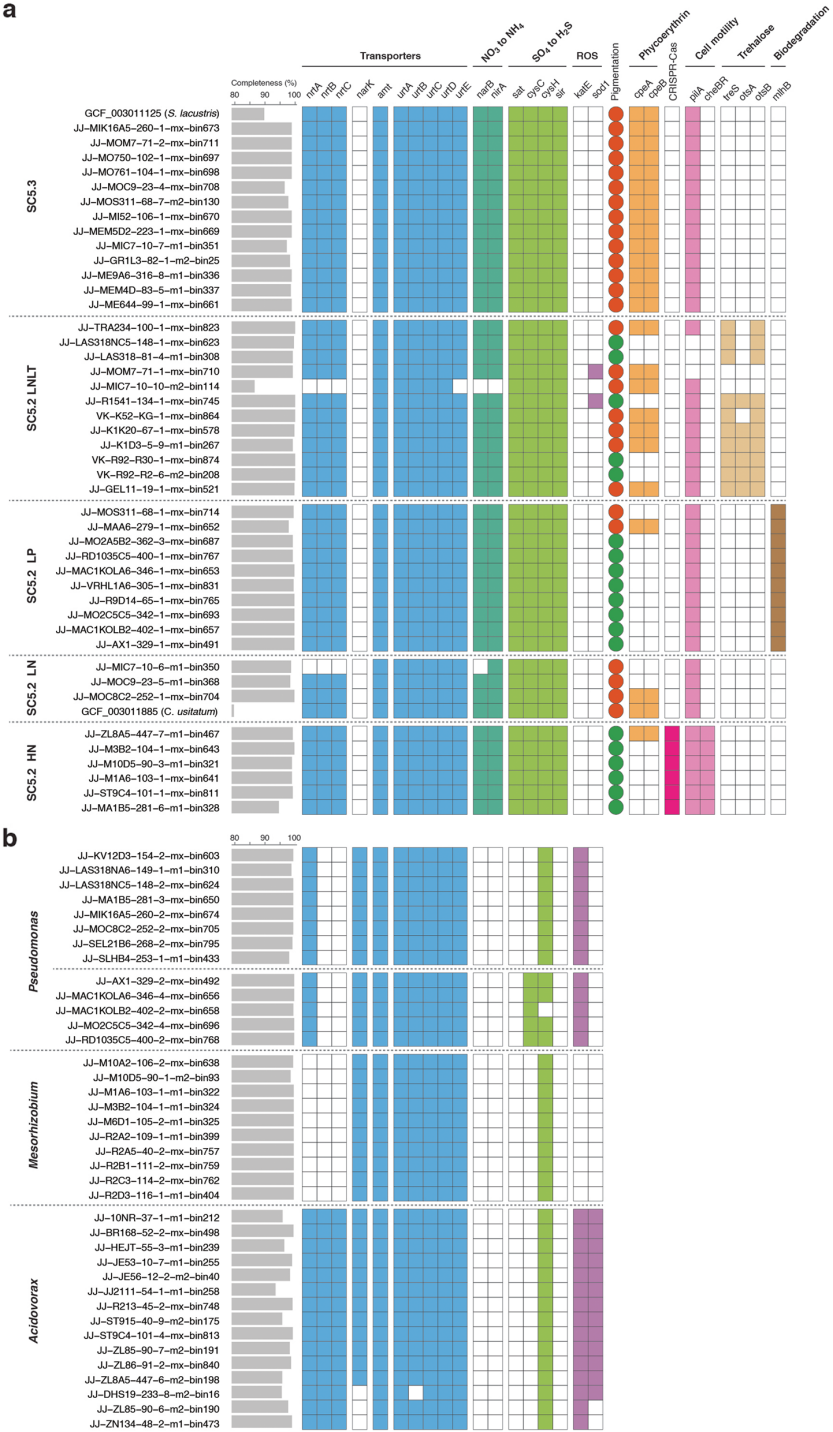
The distribution of functional genes related to symbiotic interactions and niche adaptations.** a** Picocyanobacterial genomes selected from different ecotypes. **b** The genomes of 38 heterotrophic bacteria. Empty squares symbolize the lack of the genes, while the filled squares represent the presence of the genes. In terms of pigmentation, green and red represent the cell colors of individual strains. Details of these genes are provided in the main text and Table S6–7

The potential metabolic interactions between these hetero- and photoautotrophs were next explored by analyzing their genomes (Fig. 3). The majority of the picocyanobacterial genomes encoded genes for nitrate (*narB*) and nitrite (*nirA*) reductases to convert nitrate, the sole nitrogen source in the growth media, into ammonia. They also encoded *nrtABC* for the transportation of extracellular nitrate inside the cell. On the contrary, the absence of *narB* and *nirA* in all co-occurring heterotrophs, coupled with the presence of ammonia transporters (*amt*), suggested that picocyanobacteria are likely to provide chassis for nitrogen metabolism in the community. It is also noteworthy that urea transporters were detected in both picocyanobacteria and heterotrophs. Heterotrophic bacteria further appeared to rely on picocyanobacteria for sulfur assimilation based on their genome composition. Sulfate was the primary sulfur source in the growth media, and only picocyanobacteria had the complete set of genes required to reduce sulfate into sulfide. Most heterotrophic symbionts lacked these genes, except phosphoadenosine phosphosulfate reductase (*cysH*), which catalyzes the formation of sulfite from phosphoadenosine 5′-phosphosulfate.

Heterotrophic bacteria also appeared to provide beneficial functions to picocyanobacteria in return (Fig. 3). In line with earlier marine studies [52], most picocyanobacterial genomes lacked genes necessary for detoxifying ROS (Fig. 3a). Heterotrophic bacteria showed high potential for hydrogen peroxide metabolism, with the *Pseudomonas* strains possessing up to five copies of catalase (*katE*) genes (Table S7). The genomes of *Acidovorax* were further equipped with superoxide dismutase (*sod1*) genes that control the toxic levels of ROS [53]. However, the genomes belonging to the genus *Mesorhizobium* completely lacked these antioxidant systems, leaving their complementary roles unresolved.

### Genotypic and phenotypic diversifications among distinct ecotypes

**Fig. 4:**
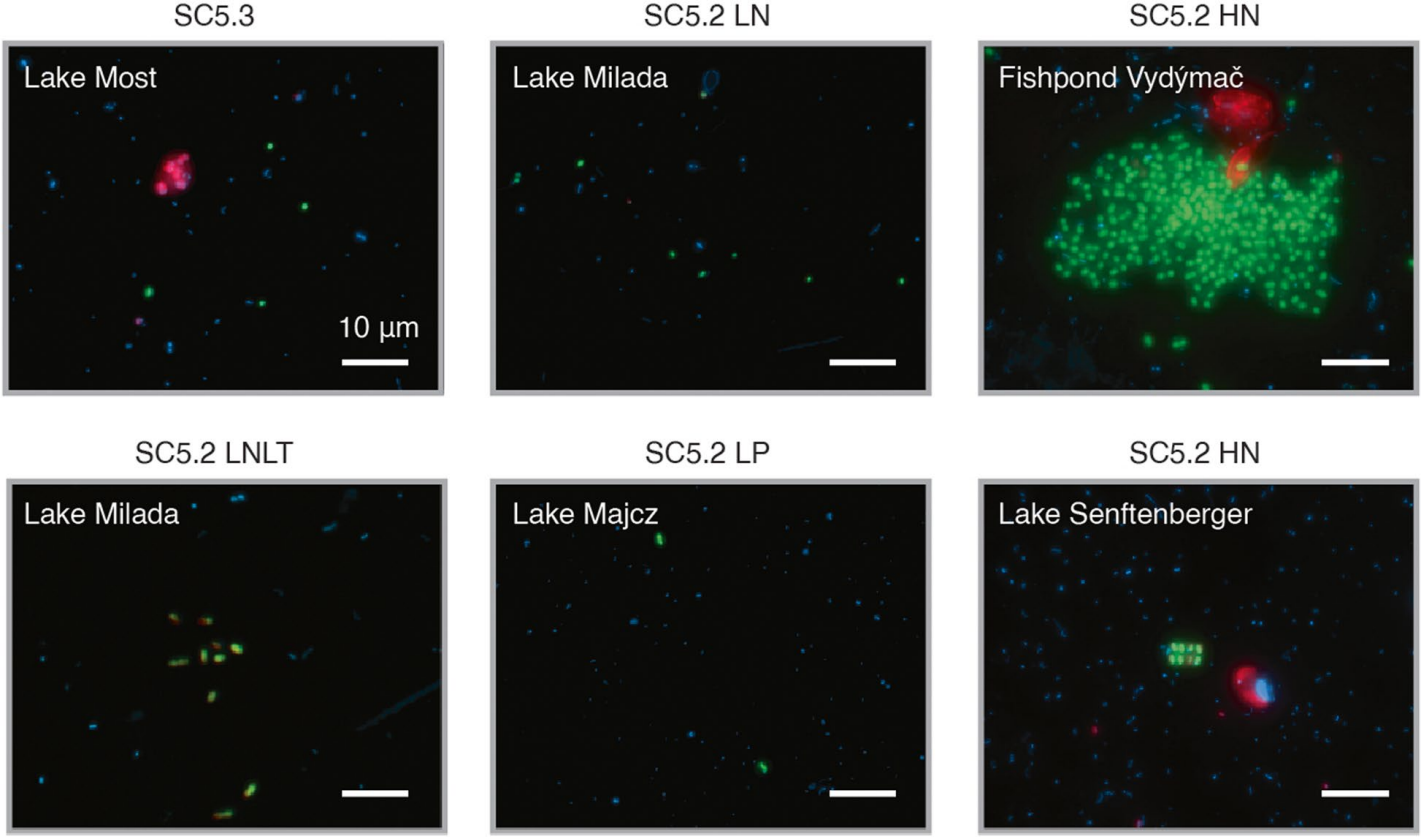
CARD-FISH images of different picocyanobacterial ecotypes. The panels display the overlap of the probe (green), DAPI (blue), and autofluorescence (red) signals

We examined the genotypic and phenotypic variations among different picocyanobacterial ecotypes to uncover the key factors contributing to their success in a wide range of ecological settings. In accordance with earlier studies [3], most picocyanobacterial populations (SC5.3, SC5.2 LN, and SC5.2 LNLT) adopting an oligotrophic lifestyle were red-pigmented, whereas the majority of the remaining isolates displayed green color (Fig. 3a and Table S2). The genomes of the red strains were set apart from the others by the presence of *cpeA* and *cpeB* genes in their genomes, which are responsible for the phycoerythrin biosynthesis. We also employed the CARD-FISH technique to characterize the growth forms of distinct ecotypes within their natural habitats (Fig. 4). In (hyper-)eutrophic waterbodies, SC5.2 HN displayed a tendency to form microcolonies or large aggregates, potentially recognized as grazing-resistant forms [16]. Conversely, the remaining ecotypes predominantly exhibited unicellular phenotypes.

A non-metric multidimensional scaling (NMDS) analysis based on the presence or absence of Kyoto Encyclopedia of Genes and Genomes (KEGG) annotated genes revealed distinct clustering of the genomes corresponding to their respective ecotypes (Fig. S5). A notable genomic variation was the selective presence of a two-component system for chemotaxis (*cheBR*) in SC5.2 HN (Fig. 3a). The presence of *pilA* encoding type IV pilus further suggests that pili-mediated chemotaxis may facilitate their ability to locate optimal conditions for growth and photosynthesis. The genomes of SC5.2 LNLT, on the other hand, harbored *treS*, *otsA*, and *otsB* genes responsible for trehalose biosynthesis. Trehalose is often recognized for enhancing photosynthesis for cyanobacteria under cold stress [54], thereby aligning well with their prevalence in cold environments. Finally, SC5.2 LP was distinguished from other ecotypes by possessing an epsilon-lactone hydrolase (*mlhB*), potentially indicating their additional capability to degrade recalcitrant humic compounds containing lactone groups [55].

### CRISPR-Cas systems in picocyanobacteria

**Fig. 5:**
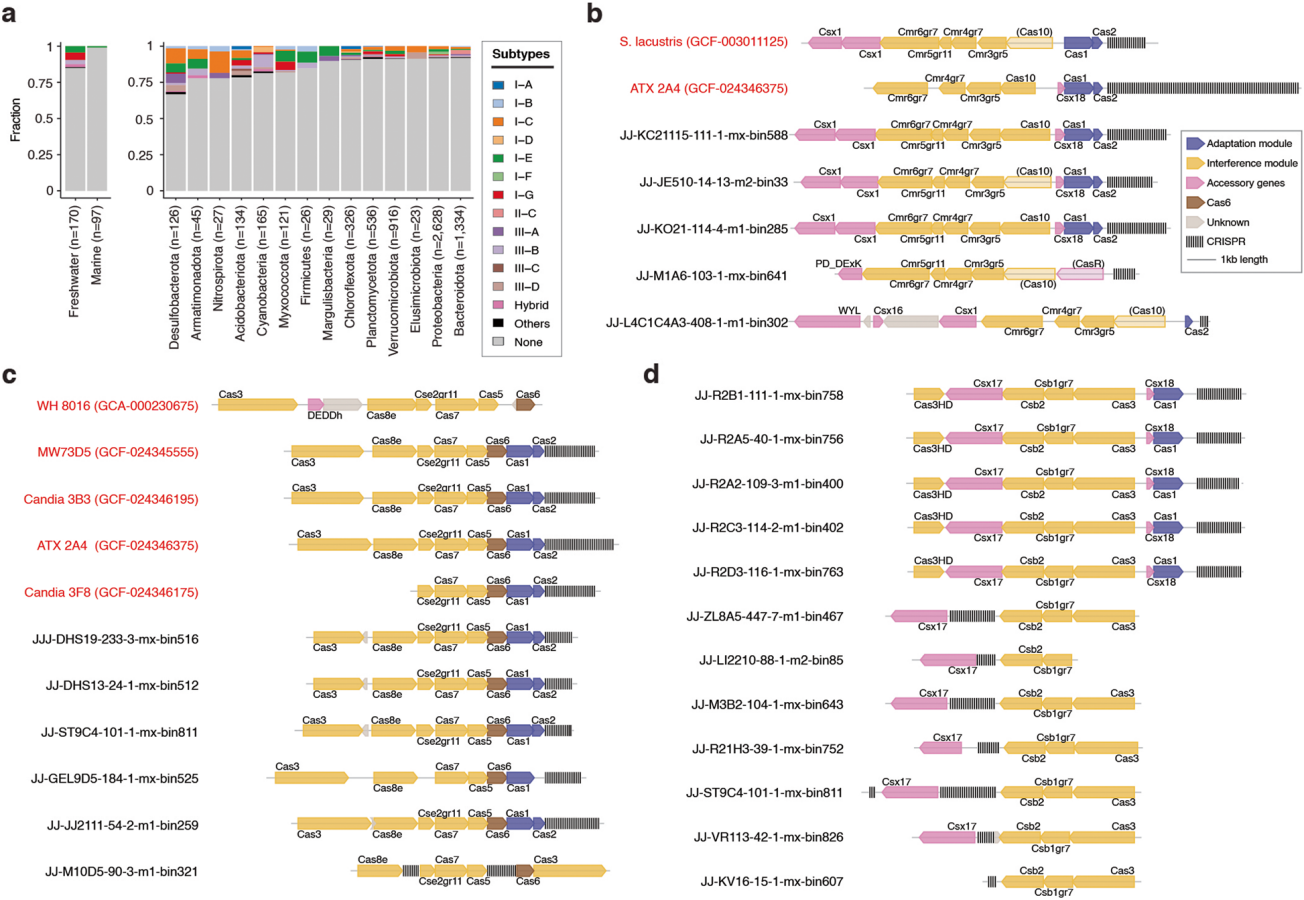
CRISPR-Cas systems in picocyanobacteria. **a** CRISPR-Cas systems in freshwater and marine picocyanobacteria (left) and the distribution of CRISPR-Cas systems among various freshwater bacterial phyla (right). Gene organization of subtype III-B (**b**), I-E (**c**), and I-G (**d**) CRISPR-Cas systems were compared for selected picocyanobacterial genomes. Genes with low-quality matches are shown in lighter shades. The genomic fragments are aligned along the cas1 genes. Publicly available picocyanobacterial genomes are colored in red

We thoroughly reassessed CRISPR-Cas systems in marine and our newly acquired freshwater genomes. As expected, CRISPR-Cas systems were absent in nearly all marine genomes (*n* = 98) examined (Fig. 5a, left panel). Conversely, our examination of the freshwater dataset revealed 15.9% (*n* = 27) of genomes encoding CRISPR-Cas systems. Further analysis indicated that CRISPR-Cas systems were found in all genomes belonging to SC5.2 HN, which were dominant in eutrophic or hypertrophic environments (Fig. 3a). On the contrary, these antiviral defense mechanisms were absent in other ecotypes, suggesting a clear segregation in the distribution of CRISPR-Cas systems based on trophic status. While previous freshwater and marine studies identified only subtypes I-E and III-B in picocyanobacterial genomes, our analysis of the newly obtained genomes has revealed the presence of subtype I-G as well. The gene organization within CRISPR-Cas operons was found to be similar between publicly available genomes and those newly obtained in this study (Fig. 5b–d). CRISPR-Cas systems consist primarily of two essential modules: an adaptation module for acquiring spacers from short segments of foreign DNA, and an interference module that recognizes and cleaves target DNA sequences [56]. Most picocyanobacterial genomes appeared to possess all the necessary components for interference modules. However, a notable fraction (40.7%) of these genomes lacked the cas1 genes required for spacer acquisition (Table S8). Interestingly, genomes lacking cas1 genes often still maintained spacers within the operon (Fig. 5b–d).

**Fig. 6:**
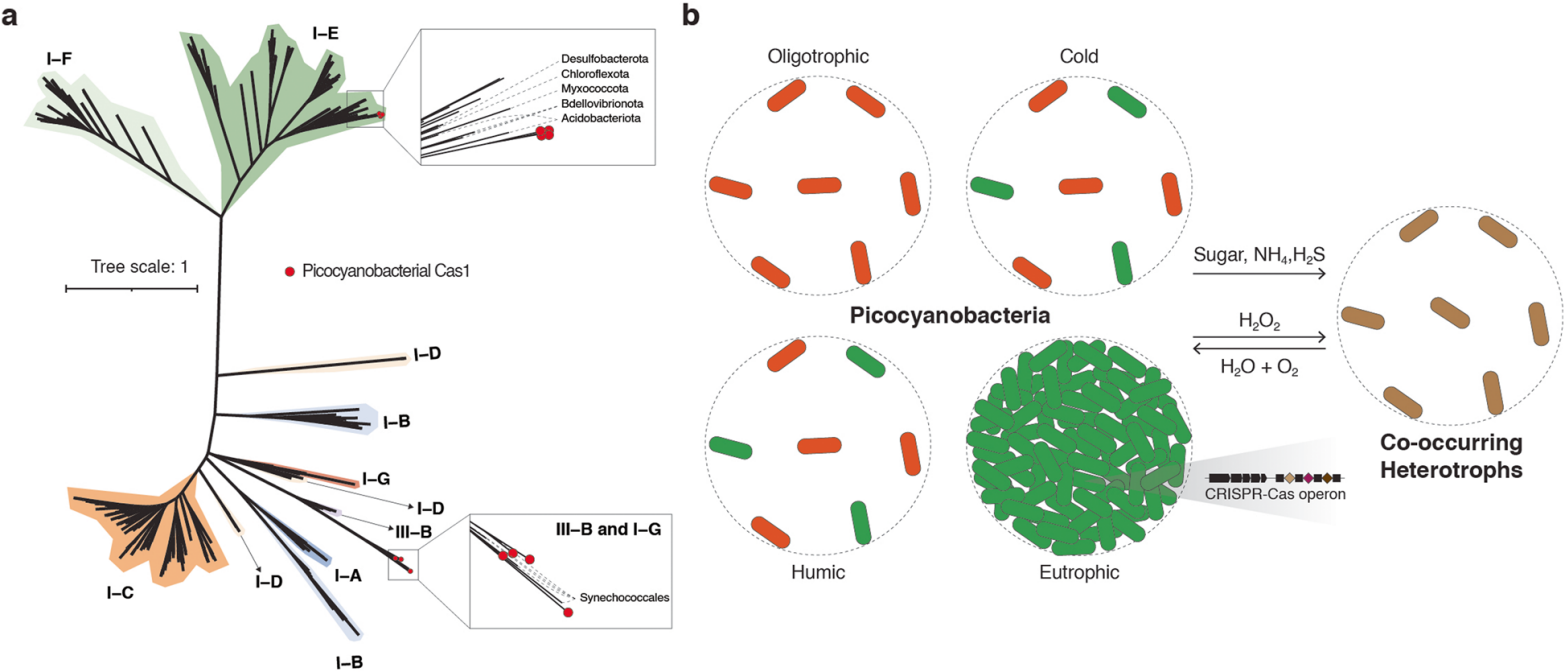
Maximum likelihood phylogenetic tree of Cas1 (**a**) and a schematic view of symbiotic interactions and niche adaptations in picocyanobacteria (**b**). **a** Picocyanobacterial Cas1 sequences are indicated by red circles at node tips. Ultrafast bootstrap values are displayed at selected nodes. Different CRISPR-Cas subtypes are represented by distinct colors. **b** Picocyanobacteria are represented in red or green depending on their pigmentation. Co-occurring heterotrophic bacteria are presented in brown. The degree of cell aggregation distinguishes the four ecologically significant ecotypes into unicellular or colonial lifestyles. The symbiotic interactions between picocyanobacteria and heterotrophs are illustrated with arrows indicating the direction of the interactions

Among cyanobacteria, subtype I-D is the most common CRISPR-Cas system, followed by I-A, III-A, and III-B [19]. Therefore, we hypothesized that the evolutionary origin of CRISPR-Cas for picocyanobacteria may involve horizontal gene transfer from different taxonomic groups. Examining a large collection (*n* = 9374) of freshwater planktonic bacterial and archaeal genomes [48] revealed that the subtypes I-C and I-E are the two most common CRISPR-Cas systems within the entire freshwater ecosystems (Fig. 5a, right panel). Among different phyla, Desulfobacterota exhibited the highest enrichment of CRISPR-Cas systems, with 30% of the genomes encoding these antiviral defense mechanisms. We investigated the evolutionary origin of the picocyanobacterial CRISPR-Cas systems through a phylogenetic reconstruction of Cas1 from all available sources (Fig. 6a). The phylogenetic tree structure of Cas1 showed a considerable agreement with the subtype classification of CRISPR-Cas, as previously described [57]. Interestingly, the subtype III-B and I-G from picocyanobacteria and other Synechococcales genomes appeared to form a clade distinct from other systems. For the subtype I-E, picocyanobacterial Cas1 clustered with orthologues from many different bacterial phyla such as Acidobacterota, Bdellovibrionota, Myxococcota, and Chloroflexota, making them potential donors.

## Discussion

Our extensive and well-coordinated sampling campaign, combined with non-axenic cultures, substantially augmented the genomic repertoire of freshwater picocyanobacteria. To our knowledge, this dataset represents the largest collection of genomes for freshwater picocyanobacteria to date, expanding the number of available genomes by more than threefold [5, 13]. Our dataset further stands out from earlier studies by employing an unbiased sampling approach that targets a variety of freshwater ecosystems (Fig. 1a). As a result, a significant portion of the genomes were acquired from eutrophic and hyper-eutrophic conditions, where the genomic availability was previously limited (Fig. 1b). However, it should be noted that while picocyanobacteria represent a major component of primary production in oligotrophic conditions, their ecological significance in eutrophic conditions might not be the same. The global phylogeography of freshwater picocyanobacteria in this study highlighted the pivotal roles of trophic status, temperature, and pH as key environmental factors delineating the major ecotypes (Fig. 1c).

The 38 genomes of the co-occurring heterotrophs isolated in this study are affiliated with four genera: *Pseudomonas*, *Mesorhizobium*, *Acidovorax*, and *Hydrogenophaga* (Fig. 2). Although their symbiotic roles for cyanobacteria have rarely been explored, all four genera have been previously observed in the cultures of filamentous cyanobacteria, such as *Microcystis* and *Anabaena* [20, 58, 59]. It has been shown that certain *Pseudomonas* species have an enhancing effect on cyanobacterial growth [20]. Our dataset further suggested that their inability to use nitrate and sulfate for growth makes them highly dependent on picocyanobacteria, presumably benefiting from carbon compounds, ammonia, and sulfide exported by their symbiotic partners (Fig. 6b). Previous marine studies on *Synechococcus* and *Prochlorococcus* demonstrated that one crucial symbiotic function of heterotrophic bacteria within their immediate environment is detoxifying ROS produced during photosynthesis [21, 25]. Within freshwater environments, the contrasting distribution patterns of genes for ROS removal between picocyanobacteria and heterotrophs support a similar conclusion that the decrease in oxidative stresses is facilitated by these heterotrophs (Fig. 6b). Yet, the interactions between cyanobacteria and heterotrophs can be far more complex since they might change over time [52] or under different growth conditions [58]. The mutualism might also occur beyond the two-species framework [60], which makes it even more challenging to understand the processes among multiple organisms.

The observed genotypic and phenotypic diversifications among defined picocyanobacterial ecotypes in this study hinted at their adaptive strategies in distinct environmental conditions (Fig. 3a). The colonial lifestyle of SC5.2 HN could be interpreted as a defense against grazing pressure (Fig. 4). What was also intriguing about this ecotype was the presence of genes encoding type IV pilus (*pilA*) and chemotaxis (*cheBR*) (Fig. 3a). While picocyanobacteria generally lack flagellar structures for motility, it has been shown that certain *Prochlorococcus* and *Synechococcus* strains rely on type IV pili to avoid sinking and predation [61].

In this study, we demonstrated the strong association between CRISPR-Cas systems and picocyanobacteria thriving in eutrophic and hyper-eutrophic conditions (Fig. 3a), which poses a fascinating contradiction to their evolutionary trajectory towards genome streamlining and reduced metabolic complexity. This observation can be potentially attributed to elevated viral loads in eutrophic lakes compared to their counterparts in oligotrophic waterbodies [62]. A strong association between high viral abundance and the increased prevalence of CRISPR-Cas systems has been previously described [63]. A recent study also demonstrated a much higher virus-to-microbe ratio in freshwater compared to marine environments [63], which could explain the scarcity of CRISPR-Cas systems in the open ocean. The CRISPR-Cas systems identified in freshwater picocyanobacteria exhibited unique phylogenetic placements and subtype classifications, setting them apart from those observed in other cyanobacteria (Fig. 6a). This observation implies an extensive evolutionary history of horizontal transfers of CRISPR-Cas loci between various taxonomic groups in freshwater ecosystems. It is important to note that a notable proportion of picocyanobacterial CRISPR-Cas systems were found to lack cas1 genes (Tabls S7). As previously described in other cyanobacteria [19], the loss of cas1 genes may represent an early stage of losing a CRISPR-Cas system. To understand how certain strains acquired CRISPR arrays without an adaptive module, a thorough investigation of viral agents and other mobile genetic elements targeting these strains will be necessary.

## Conclusions

Together, our findings effectively addressed gaps in understanding population diversity, symbiotic interactions, and adaptation strategies of picocyanobacteria within freshwater environments. Furthermore, this work establishes a genomic foundation for future efforts aimed at the detailed characterization of the genomic landscapes of freshwater picocyanobacteria, in connection with their evolutionary trajectories.

### Supplementary Information

Supplementary Material 1. Figure S1. Geographic distribution of the sampling sites across Central Europe. The inset at the top left shows the entire Europe, with sampled countries colored in yellow. Two letter codes represent the respective countries. Figure S2. Full phylogenomic tree of picocyanobacterial genomes. Maximum likelihood phylogeny of 170 new isolates from this study and additional 79 publicly available genomes. Prochlorococcus genomes (n=16) were used to root the tree. Figure S3. Genome sizes and % GC for different picocyanobacterial ecotypes. Figure S4. A heatmap of average nucleotide identity (ANI) values across freshwater picocyanobacterial genomes. Genomes are ordered in accordance with the phylogenomic tree. Figure S5. NMDS analysis based on the presence and absence of KEGG genes.

Supplementary Material 2. Table S1. Sampling sites across the central Europe. Table S2. Genome statistics, culture conditions, and pigmentation phenothypes of picocyanobacteria. Table S3. Global distribution of freshwater picocyanobacteria calculated by metagenomic read recruitment (base coverage per Gb). Table S4. List of 526 heterotrophic bacterial genomes co-isolated in the cultures. Table S5. List of 38 heterophic bacterial genomes present in more than 5% of the cultures. Table S6. KEGG annotation results of picocyanobacterial genomes. Table S7. KEGG annotation results of heterotrophic partners. Table S8. CRISRP-Cas prediction from picocyanobacterial genomes using the CRISPRCasTyper. Table S9. CARD-FISH probes for different ecotypes of freshwater picocyanobacteria and the concentration of formamide in the hybridization buffer.

## Data Availability

The genome sequencing data from this study has been deposited in EBI ENA under the Bioproject PRJEB49198. Additional data, such as individual fasta files for picocyanobacterial and heterotrophic bacterial genomes, alignment files, and trees are available in Figshare (https://doi.org/10.6084/m9.figshare.24105627.v1).

## Abbreviations

CRISPR-Cas: Clustered regularly interspaced short palindrome repeats-CRISPR associated proteins
ROS: Reactive oxygen species
ANI: Average nucleotide identity
LN: Low-nutrient
LNLT: Low-nutrient and low-temperature
HN: High-nutrient
LP: Low-pH
CARD-FISH: Catalyzed reporter deposition-fluorescence in situ hybridization
NMDS: Non-metric multidimensional scaling
KEGG: Kyoto Encyclopedia of Genes and Genomes
