# Supplementary material for: Uncovering the genomic basis of symbiotic interactions and niche adaptations in freshwater picocyanobacteria
Source: Microbiome. 2024 Aug 10;12:150. doi: 10.1186/s40168-024-01867-0 (PMC11316352; doi:10.1186/s40168-024-01867-0)
Supplement: Supplementary file 2 — Supplementary Material 1. Figure S1. Geographic distribution of the sampling sites across Central Europe. The inset at the top left shows the entire Europe, with sampled countries colored in yellow. Two letter codes represent the respective countries. Figure S2. Full phylogenomic tree of picocyanobacterial genomes. Maximum likelihood phylogeny of 170 new isolates from this study and additional 79 publicly available genomes. Prochlorococcus genomes (n=16) were used to root the tree. Figure S3. Genome sizes and % GC for different picocyanobacterial ecotypes. Figure S4. A heatmap of average nucleotide identity (ANI) values across freshwater picocyanobacterial genomes. Genomes are ordered in accordance with the phylogenomic tree. Figure S5. NMDS analysis based on the presence and absence of KEGG genes. [file 40168_2024_1867_MOESM1_ESM.pdf]

Uncovering the genomic basis of symbiotic interactions and niche adaptations in freshwater picocyanobacteria

Hongjae Park[1*†], Paul Bulzu[1*], Tanja Shabarova[1], Vinicius S. Kavagutti[1], Rohit Ghai[1], Vojtěch Kasalický[1], and Jitka Jezberová[1]

[1]Institute of Hydrobiology, Biology Centre of the Czech Academy of Sciences, České Budějovice, Czech Republic

[*]These authors contributed equally to this work

[†]Corresponding author: park.hongjae0@gmail.com

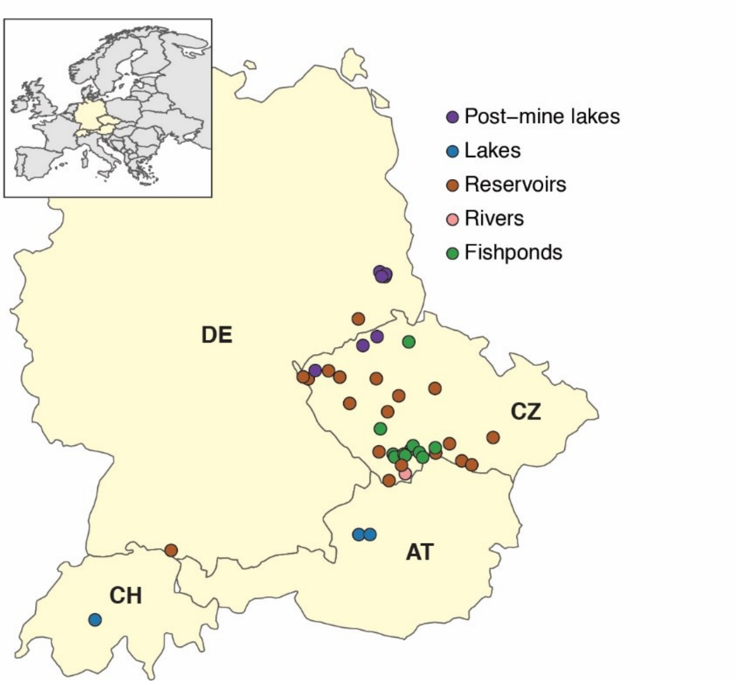

Figure S1. Geographic distribution of the sampling sites across Central Europe. The inset at the top left shows the entire Europe, with sampled countries colored in yellow. Two-letter codes represent the respective countries.

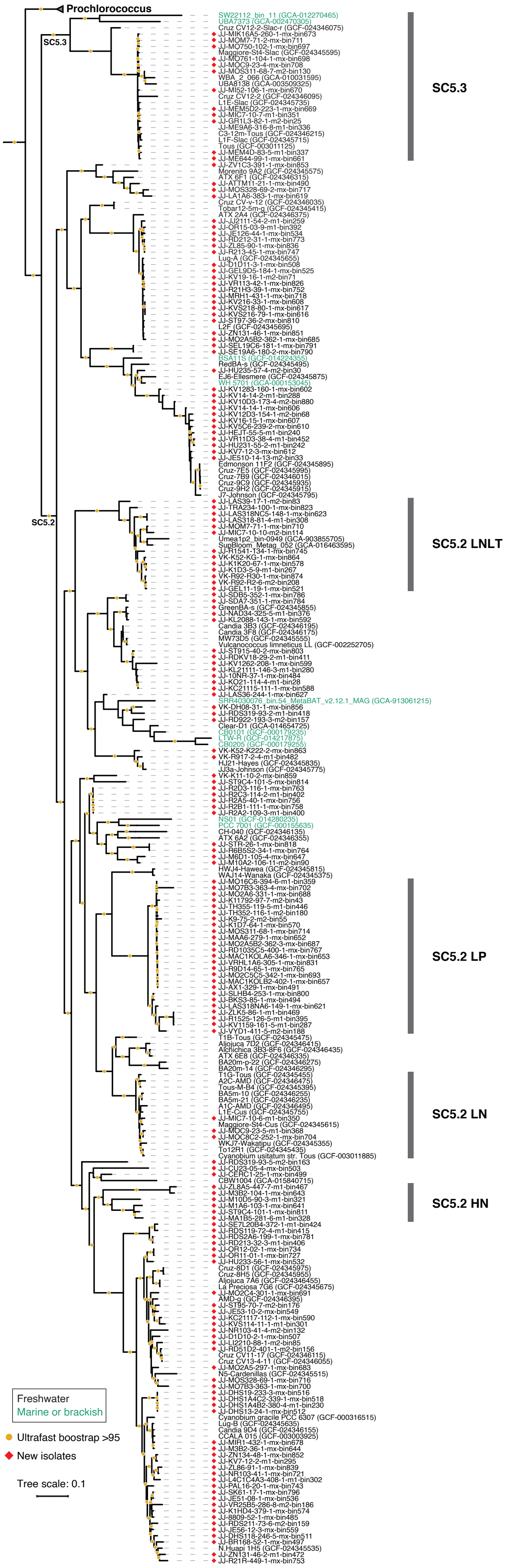

Figure S2. Full phylogenomic tree of picocyanobacterial genomes. Maximum likelihood phylogeny of 170 new isolates from this study and additional 79 publicly available genomes. Prochlorococcus genomes (n=16) were used to root the tree.

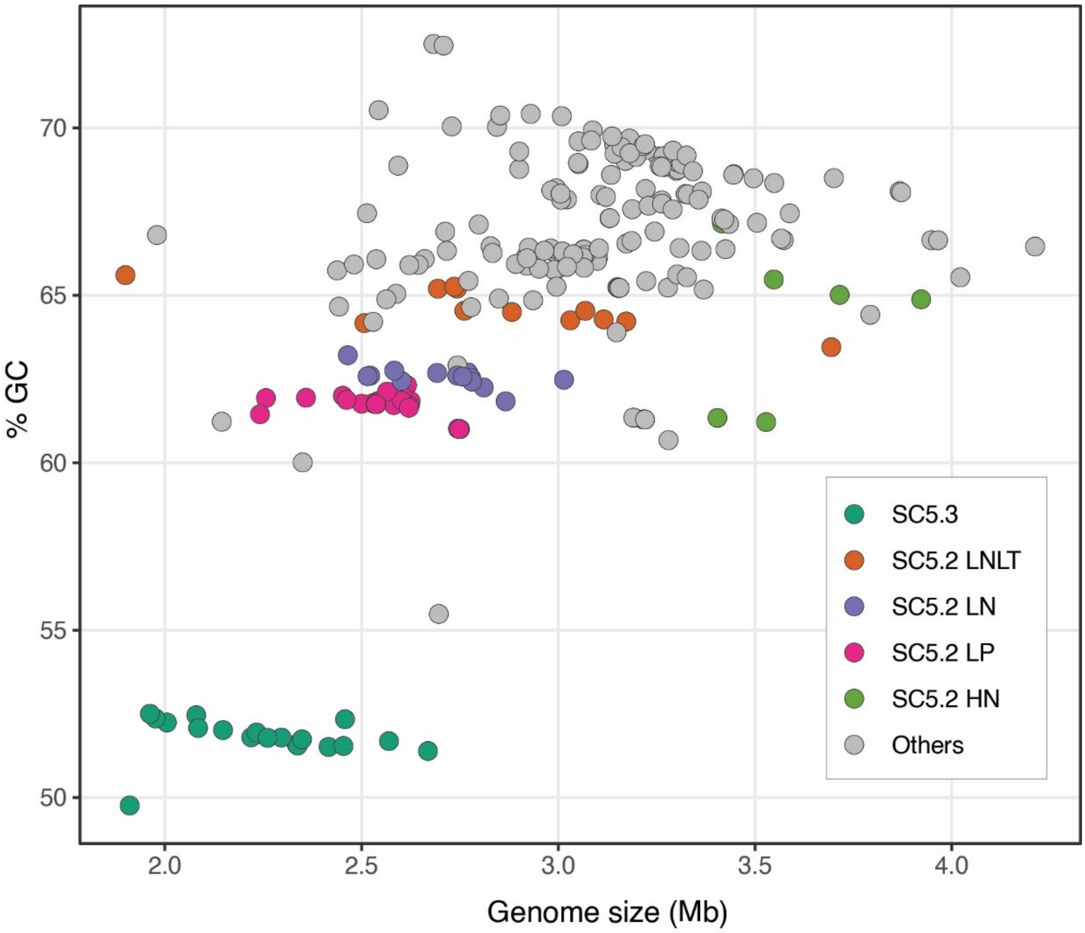

Figure S3. Genome sizes and % GC for different picocyanobacterial ecotypes.

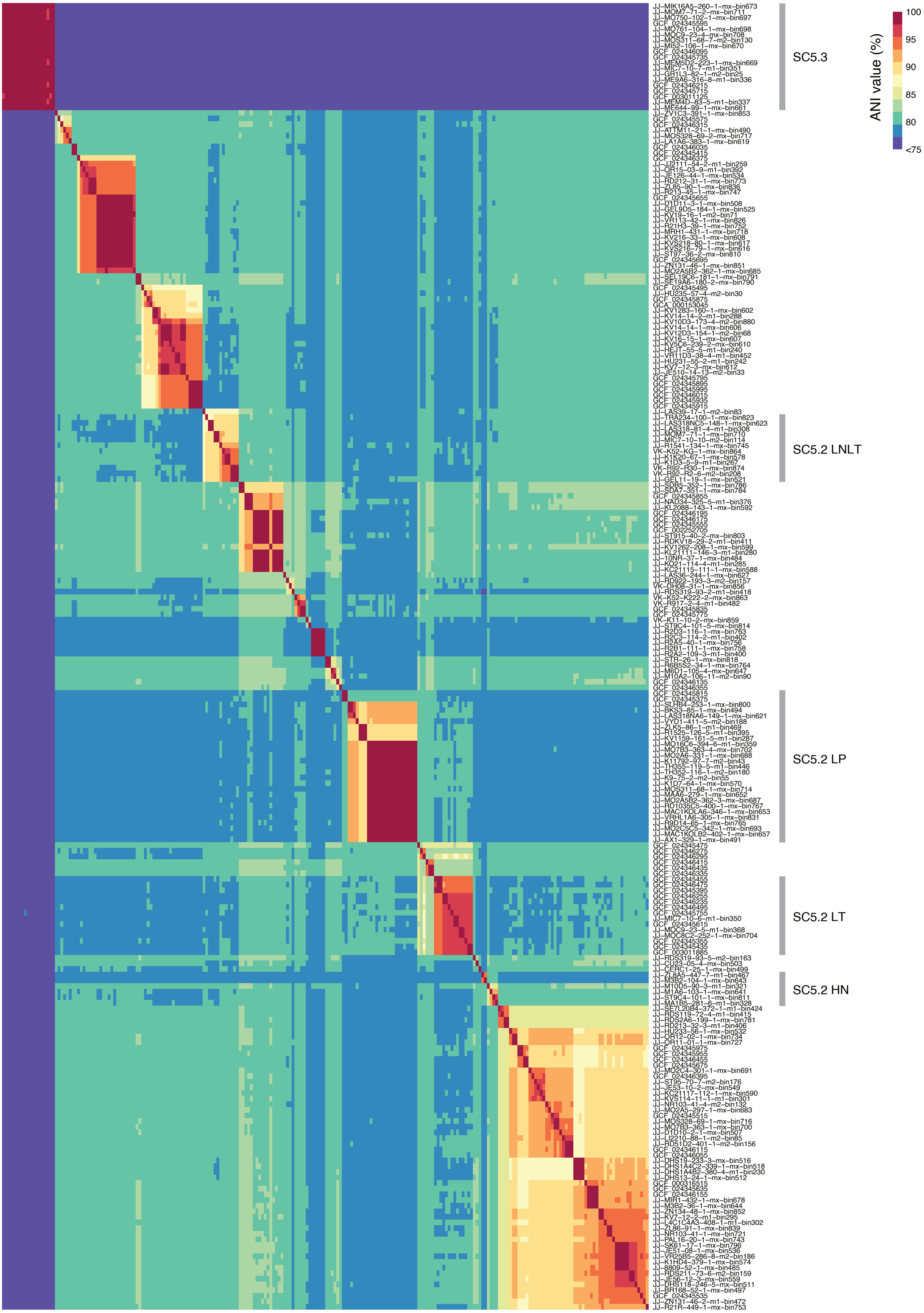

Figure S4. A heatmap of average nucleotide identity (ANI) values across freshwater picocyanobacterial genomes. Genomes are ordered in accordance with the phylogenomic tree.

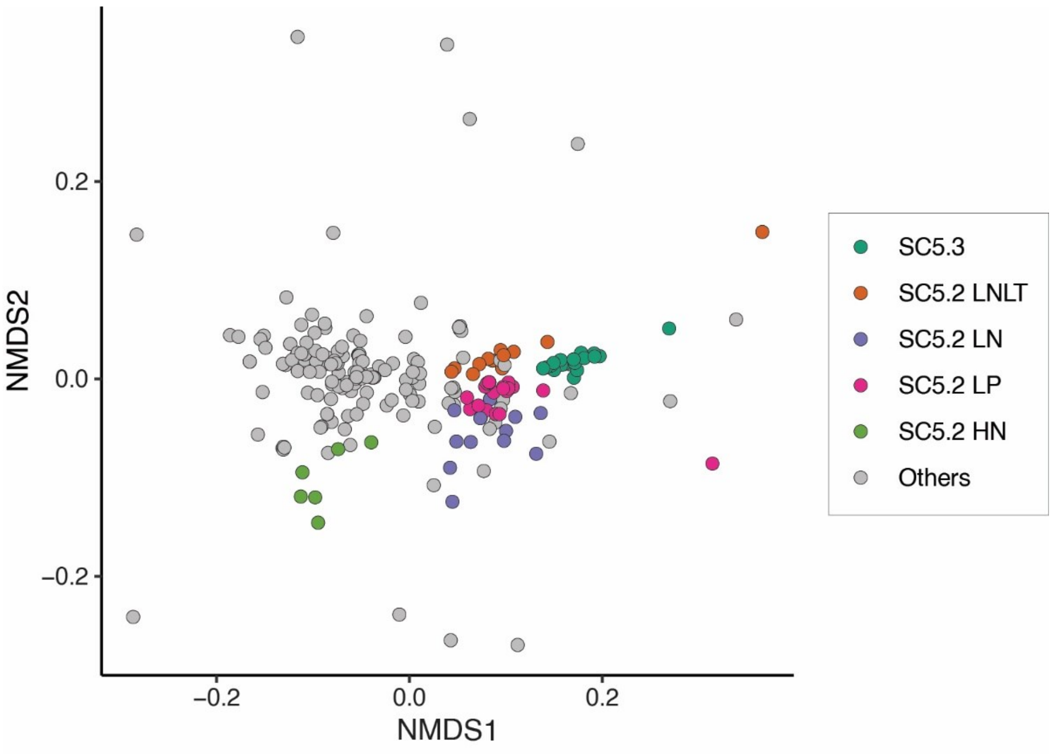

Figure S5. NMDS analysis based on the presence and absence of KEGG genes.
